# Risk identification strategies for health pandemics and epidemics on college campuses: A comprehensive analysis of heat maps and behavioral observations

**DOI:** 10.1371/journal.pone.0343811

**Published:** 2026-03-16

**Authors:** Matthew M. Laske, Abigail L. Blackman, Fernanda S. Oda, Derek D. Reed, Florence D. DiGennaro Reed

**Affiliations:** Department of Applied Behavioral Science, University of Kansas, Lawrence, Kansas, United States of America; Nanyang Technological University, SINGAPORE

## Abstract

**Objective:**

The purpose of the current studies was to use direct observation and surveys to assess behavioral risk on a college campus during the COVID-19 pandemic.

**Methods:**

Study 1 used direct observation to monitor safe and at-risk mask-wearing behavior across multiple campus locations and whether mask-wearing differed when individuals were alone or in groups. Study 2 surveyed college students through a click-based campus map survey to identify high- and low-mask-wearing locations on campus and create data heat maps indicating at-risk behaviors. Direct observation analyses were then used to verify the identified locations.

**Results:**

Study 1 revealed that mask-wearing was likely during a mask-wearing policy implemented across the college campus. However, mask-wearing was less likely when people were in groups than alone. Study 2 showed that survey responses and the heat map analysis identified spatially distinct locations of perceived high and low mask-wearing. These data were validated through direct observation, verifying high and low mask-wearing at the identified locations.

**Conclusions:**

Survey heat maps paired with direct observation can help identify specific locations where safe and at-risk behaviors are most likely to occur. By identifying special patterns of risk, this comprehensive approach offers actionable information to guide interventions, policy enforcement, and resource allocation during health epidemics.

## Introduction

Over 900,000 nonfatal illnesses occurred in the United States workplaces between 2020 and 2021 [1]. Of those illnesses, nearly 700,000 were due to respiratory infections, such as the flu, a cold, or coronavirus disease 2019 (COVID-19). During this time, educational settings, such as universities, also saw increases in respiratory illnesses. Specifically, the rate of respiratory illnesses per 10,000 full-time employees, not including students, increased from 5.5 in 2020 to 13.5 in 2021 [1]. These data are likely linked to the COVID-19 pandemic and universities transitioning back to in-person instruction in 2021.

Many universities implemented policies and procedures to monitor and mitigate COVID-19 risk. The benefit of monitoring safe and at-risk behaviors is that they can be acted upon before the illness occurs (e.g., contracting COVID-19). During the pandemic’s peak, monitoring COVID-19 cases alone was less effective because, by the time a case was detected, the at-risk behavior leading to infection had already occurred and thus could not be prevented. The critical safe behavior for mitigating the spread of respiratory illnesses during epidemics is mask-wearing [2,3]. The at-risk alternative is not wearing a mask or wearing a mask incorrectly. During public health epidemics on college campuses, identifying locations with lower mask-wearing is particularly important given the dense living, eating, and social environments for many students. Prioritizing specific locations, days, and times can allow a university to implement more effective policies and procedures to mitigate low mask-wearing. Therefore, identifying locations where low mask-wearing behavior occurs is necessary. Throughout this manuscript, behavioral risk refers to observable patterns of mask-wearing behavior (e.g., locations with lower mask-wearing), rather than estimating infection risk at any given location.

In addition to location, mask-wearing may also vary due to social norms. For example, longitudinal research demonstrated that descriptive norms (i.e., perceptions of what others typically do) were related to self-reported mask-wearing behavior at various points during the COVID-19 pandemic [4]. Prior survey research suggests that people report lower intentions to wear masks and evaluate non-masking less critically around friends and family than around strangers [5]. These results suggest that mask-wearing may be influenced by whether people are in familiar groups or not. In university settings, social norms around mask-wearing are likewise related to reported mask-wearing behavior [6]. Together, this literature suggests that mask-wearing may differ meaningfully between individuals alone and in groups, and that it may vary across locations. To monitor these risk behaviors, it is necessary to identify where they are most likely to occur and under what social contexts and select an appropriate measurement system.

Direct observations and surveys can be used to measure behavioral risks. Direct observation involves training observers to observe and document safe and at-risk behavior. For example, Geller, Kalsher, Rudd, and Lehman [7] observed seat belt wearing on a college campus and evaluated the effects of commitment and incentive strategies. Burnell, Robbins, Kulali, and Wells [8] trained observers to count mask-wearing use at a college campus. Direct observation is the only method to verify safe and at-risk behaviors as they occur, rather than relying on self-report. A disadvantage of direct observation during health epidemics is the time required to find and verify where to concentrate observations to measure where at-risk behaviors are most likely. Therefore, surveying methods can also be used.

Surveying for risk is often done by asking respondents, typically through a questionnaire, to report safe or at-risk behaviors. For example, Wray, Hansen, Ding, and Masters [9] surveyed university students, staff, and faculty and asked them to self-report their tobacco use before and after the implementation of a policy. A benefit of surveys is that they require less effort to implement compared to direct observation. However, survey responses are a proxy for safe and at-risk behavior and require verification.

Surveys can also be used to capture specific locations where safe and at-risk behaviors occur. One way to do this is through heat mapping, where survey respondents can click locations on a map, and the clicks can be aggregated and visualized on a map. Heat mapping visuals can then be used to identify locations where safe and at-risk behaviors are reported. For example, Gelino, Salzer, Harsin, Naudé, Gilroy, and Reed [10] surveyed a university’s faculty, staff, and students to determine areas on campus where tobacco smoking occurred. Heat mapping methods—particularly when integrated into widely available survey platforms such as Qualtrics®—offer a practical and scalable approach to identifying perceived behavioral risks across large geographic areas. These maps are easily generated from survey responses, which not only enhances face validity through visual representation but also enables rapid, large-scale data collection. Embedding such tools in routine student or staff surveys could serve as an early warning system, helping institutions identify behavioral risk hotspots in real time and deploy intervention resources more efficiently. Despite the potential use of heat mapping, self-reported safe and at-risk behavior is a proxy for the actual behavior. Therefore, validating the results of survey-based and heat-mapping methods through direct observation is necessary to confirm the measure’s efficacy [10].

The current studies used direct observation, survey, and heat mapping methods to identify higher versus lower mask-wearing locations on a college campus during the COVID-19 pandemic. In Study 1, mask-wearing was directly observed across multiple locations on a college campus and we observed whether mask-wearing differed when individuals were alone or in a group. In Study 2, students completed a click-based campus map survey to identify locations perceived as lower versus higher mask-wearing. These responses were aggregated into visual heat maps and direct observation was then used to validate mask-wearing behavior at those locations.

## Study 1

This study aimed to monitor mask-wearing across a college campus to identify patterns of behavioral risk during the COVID-19 pandemic. At the time of data collection, the university had a policy requiring mask-wearing anywhere on campus, both indoors and outdoors. Observation locations were selected based on logistical considerations. Specifically, locations were selected for observation based on high-traffic areas, such as major classroom buildings, centralized bus stops, libraries, and the student union. While these locations were selected for anticipated high traffic, they were not selected based on data-informed behavioral risk (e.g., direct observation or survey reports).

### Hypotheses

Given the university-wide mask-wearing mandate and the absence of behavioral risk data, it was hypothesized that mask-wearing would generally be relatively consistent across campus observation locations, with no meaningful differences in mask-wearing adherence by location (H1). In addition, mask-wearing behavior may vary depending on whether individuals are alone or in groups. Social factors could influence at-risk behaviors in both directions. For example, individuals walking alone may perceive lower personal risk and therefore be less likely to wear a mask. However, group social dynamics may pressure them to conform to non-mask-wearing norms if others in their group are not wearing masks [6]. Additionally, survey-based research has revealed that people are less critical of mask-wearing as a norm around friends/family and have lower intentions of wearing masks than when around strangers [5]. Therefore, individuals in a group may be less likely to wear a mask when they know the person, due to perceptions of decreased risk. H2 asked whether individuals walking in groups are less likely to wear masks than individuals walking alone.

### Methods

#### Direct observation procedures.

**Fig 1:**
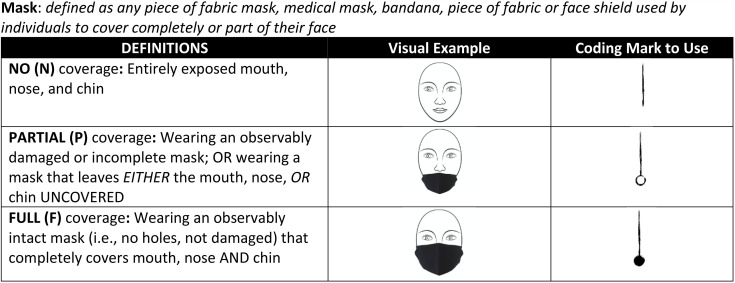
Mask-wearing scoring key. The scoring key was printed on the front of every datasheet.

Direct observations were conducted outside at various locations across the university. These observations were considered exempt from research by the university’s Human Research Protection Program because they were public observations of behavior. Observations were conducted over a 10-week period between February 10th, 2021, and April 9^th^, 2021. Trained observers conducted 30-minute observations at an assigned area. To complete the observation, observers would score mask-wearing for people who walked past a predetermined line at the location (e.g., a straight line from the observer’s location to the bus stop sign). Mask-wearing was scored as full, partial, or no wearing. Full mask wearing was scored if a person wore a fully intact mask (i.e., no holes or damage) that completely covered the mouth, nose, and chin. Partial mask wearing was scored if a person wore a damaged or incomplete mask or wearing a mask that left the mouth, nose, or chin uncovered. No mask-wearing was scored if a person did not have a mask covering their mouth, nose, and chin. Common examples of no mask wearing include wearing the mask around the neck or not having a mask present. When a person walked by the observer’s location, the observer would use a coding scheme to score each mask-wearing category. Full mask-wearing was scored using a straight line with a fully closed circle at the end, partial mask-wearing was scored using a straight line with an open circle at the end of the tally, and no mask-wearing was scored using a straight-line tally. Fig 1 contains the definitions and coding scheme used for direct observations.

#### Group status classification.

**Fig 2:**
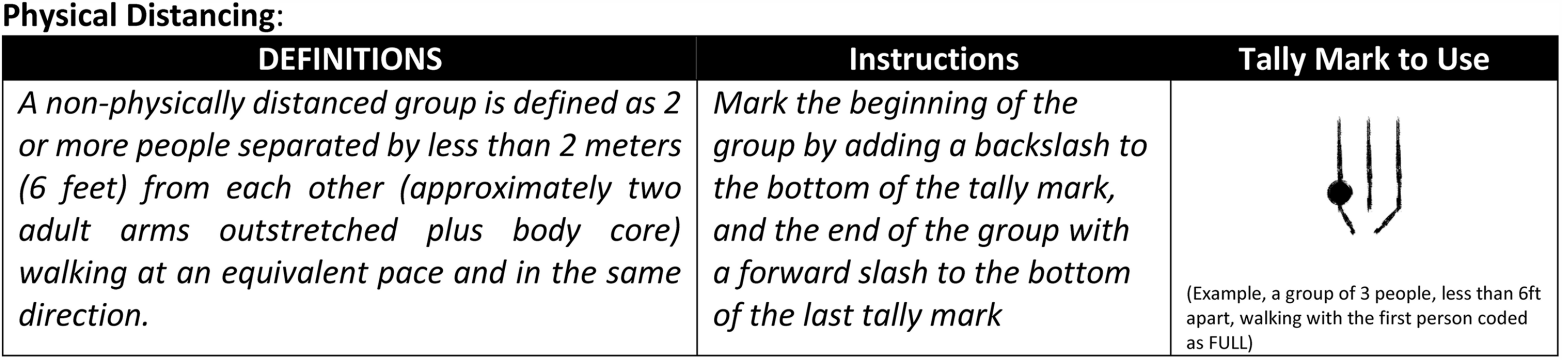
Group status scoring key. The scoring key was printed on the front of every datasheet.

A subsample of observations also included scoring whether a person was in a group or alone (i.e., whether the person was physically distanced or not). We defined a person’s group status as two or more people separated by less than 2 meters (6 feet) from each other, walking at an equivalent pace and in the same direction. To indicate group status on the observation data sheet, the observer would add a backslash to the first tally representing the start of the group and a forward slash on the last tally to indicate the end of the group. Fig 2 contains the definitions and coding scheme used for group status.

#### Interobserver agreement.

A secondary independent observer collected data for 11.32% of observations to ensure the reliability of observation data. The independent observer conducted an observation at the same time and location as the primary observer. The observers completed their observations independently of one another. For scoring purposes, full, partial, and no mask-wearing were scored as separate trials for comparison; then, interobserver agreement was calculated by averaging the percent trial agreement across a given observation and multiplying by 100. Mean agreement across all observations was high, averaging 80.60% (range, 63.18%–99.03%). These data indicate that observation scoring was generally reliable across observers.

### Results

#### Direct observation findings.

**Fig 3:**
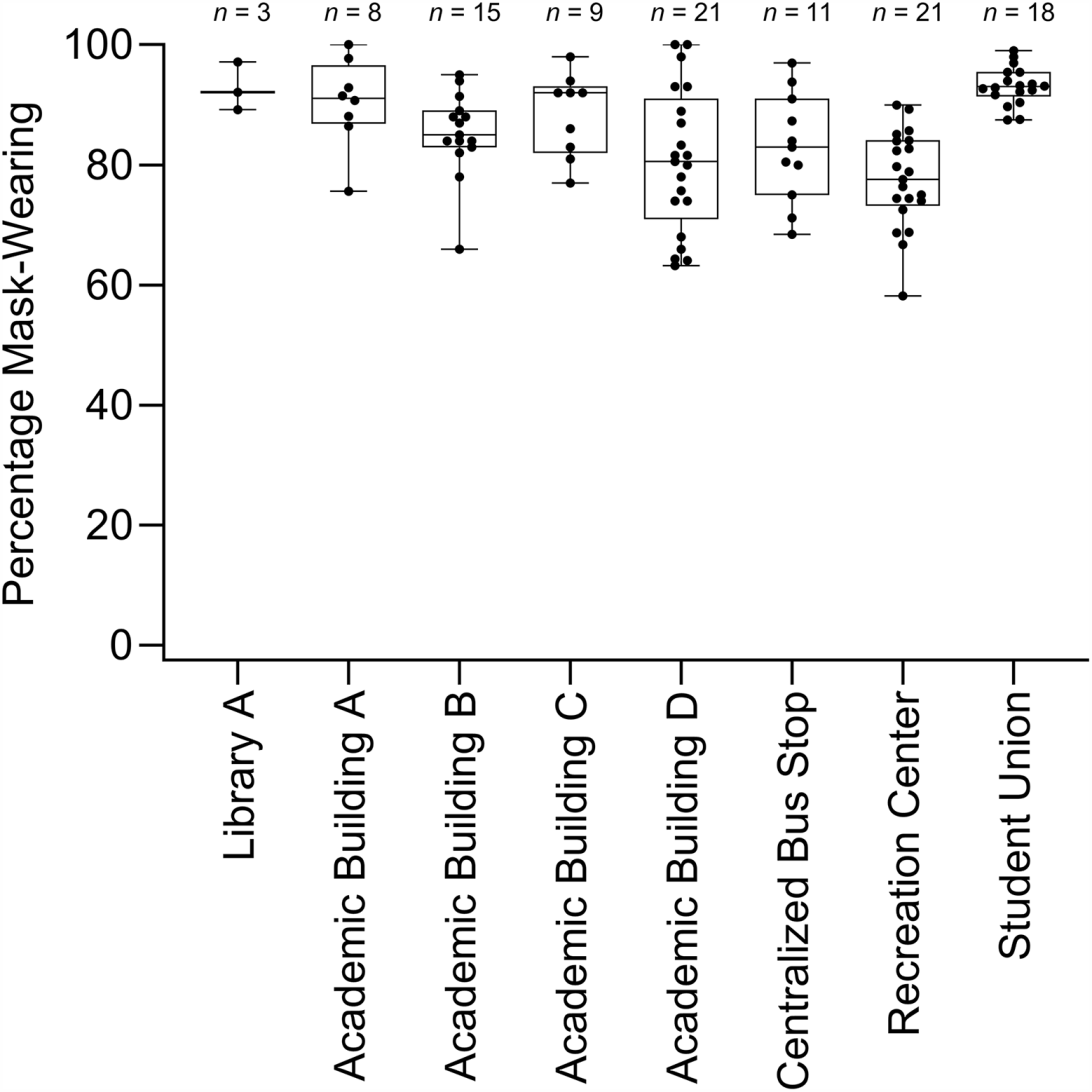
Percentage mask wearing across all locations. Box plot whiskers indicate the minimum and maximum data. The beginning and end of the box plot indicate the 25th and 75th percentile values. The horizontal line within the box plot indicates the median. N represents the number of 30 min observations conducted at that location.

Over ten weeks, 106 observations were completed. This resulted in 53 hours of observation. Fig 3 contains observations across locations and the percentage of mask-wearing observed during each observation. Results from direct observation revealed minimal variance in mask-wearing across the university. Overall, mask-wearing percentages were high, averaging 85% (range, 58%–100%) across all locations. To assess whether mask-wearing differed significantly by location, an ANOVA was conducted in GraphPad Prism® (version 10.1.2). Prior to selecting an appropriate test, the data were assessed for normality. In addition to reviewing distribution plots for normality and QQplots, a Shapiro-Wilk test assessed whether the data were normally distributed. No location had a significant Shapiro-Wilk test, indicating that each distribution passed a test for normality. Although normality assumptions were met based on Shapiro-Wilk tests for each location, a non-parametric Kruskal-Wallis test was used due to small sample sizes in some locations (e.g., 3 observations at Library A). Mask-wearing was significantly affected by the observation location, *H*(7) = 38.33, *p* < .001. Post hoc Dunn’s multiple comparisons test between locations identified several differences, although most comparisons were nonsignificant, suggesting that mask wearing was generally consistent across campus. Specifically, the only statistically significant differences between locations were between Academic Building A and the Recreation Center (*difference* = 41.03, *p* = .0369), Academic Building D and the Student Union (*difference* = −41.07, *p* = .0009), the Centralized Bus Stop and the Student Union (*difference* = −37.06, *p* = .0457), and the Recreation Center and the Student Union (*difference* = −53.05, *p* < .0001). There were no other statistically significant differences across locations. These results provide partial support for H1. Specifically, only four of the 28 location comparisons had statistically significant differences, indicating some variability across locations.

#### Group status findings.

**Fig 4:**
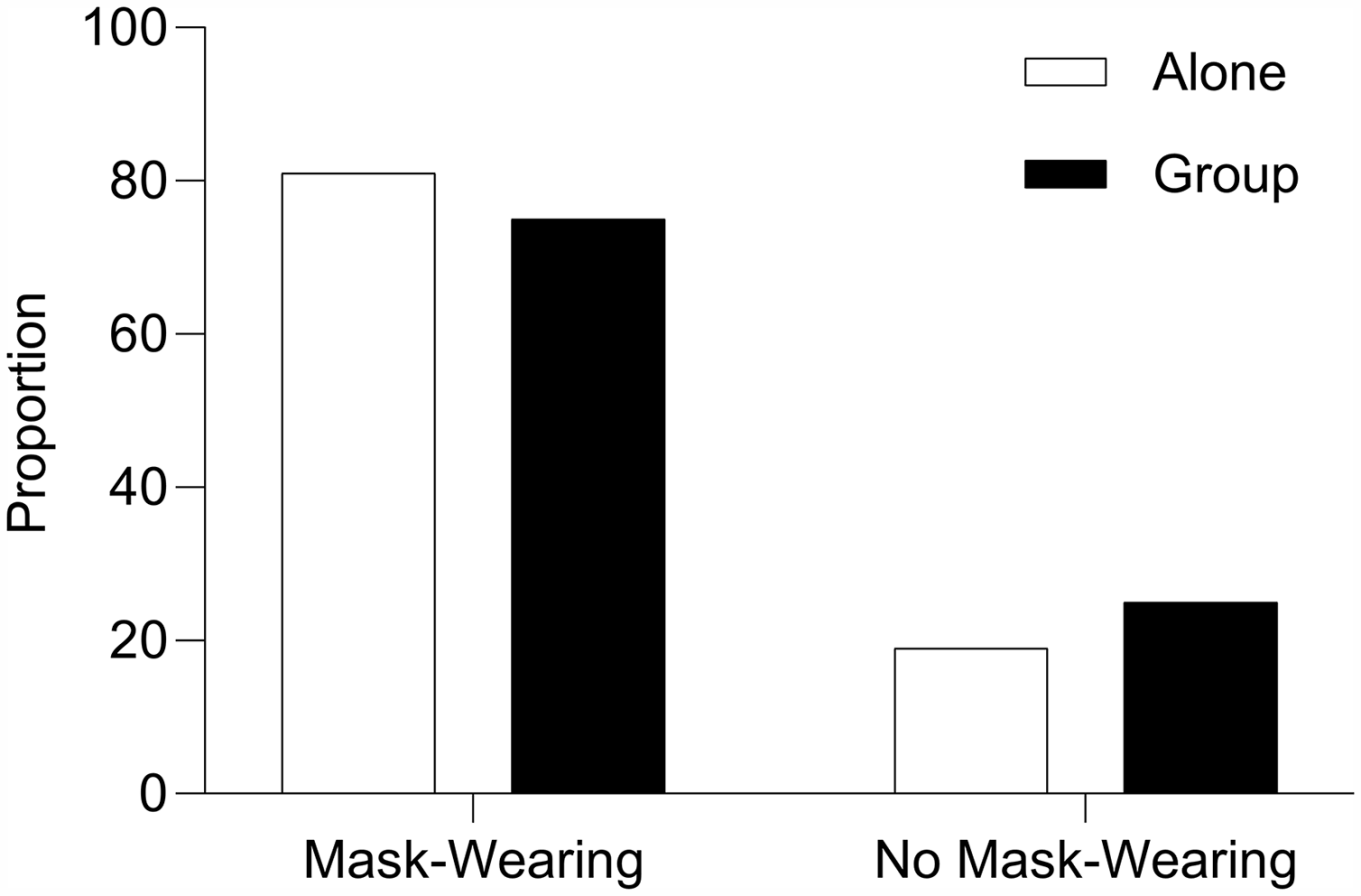
Proportion of mask-wearing by group status. Bar graph indicating the proportion of individuals mask-wearing behavior by group status. White bars represent the proportion of individuals who were walking alone. Black bars represent the proportion of individuals in a group.

To assess H2 about whether group status (i.e., walking alone vs. in a group) was associated with mask-wearing behaviors, mask-wearing and group status were scored for 8,427 people. See Fig 4 for proportions of mask-wearing among individuals walking alone versus in groups. A total of 3,496 people were observed in a group (41.5% of the total people observed). A total of 882 people in a group (25% of the total in a group) were not wearing a mask. A total of 2,614 (75% of the total in a group) in a group were wearing a mask. A total of 4,931 people were observed walking individually (58.5% of the total people observed). A total of 954 (19% of the total walking alone) walked individually without wearing a mask. A total of 3,977 (81% of the total walking alone) walked individually wearing a mask. A chi-square analysis was conducted using the CrossTable function in the gmodels library (version 2.19.1) through RStudio (version 2025.05.01). This analysis revealed a significant relation between walking alone or within a group and whether a mask was likely to be worn χ2(1) = 41.53, p < .001. By observing the odds ratio, the odds of a person not wearing a mask were 1.41 (1.27, 1.56) times more likely if the person was walking within a group versus alone. These findings suggest that the mask-wearing behavior varied systematically by group status, with individuals walking in groups being more likely to engage in at-risk behavior (i.e., not wearing mask), providing evidence of a potential social norm effect.

### Study 1 discussion

The results of this study indicated that mask-wearing was generally high across multiple locations on campus. Although some statistically significant differences were found between specific locations, most comparisons did not differ significantly, providing partial support for H1. These findings suggest that mask-wearing behavior was relatively stable across locations selected based on logistical considerations, like class schedules, bus location, and commonly high-traffic buildings (i.e., student union and libraries). These results are consistent with other literature that has reported high mask wearing at a United States campus with an indoor and outdoor mask-wearing policy [6]. Additionally, there was some variability in the percentage of mask-wearing within each location. This within-location variability could have been due to differences in time of day for observation or crowd density. For example, the total number of individuals observed in a 30 min observation ranged from seven to 216. Therefore, a smaller sample size within each observation could drastically change the percentage calculation.

In addition, group status influenced mask-wearing behavior. Our analysis supported H2 and found that being in a group predicted lower mask-wearing than walking alone. These results suggest that social norms or peer influence may reduce mask adherence at a university campus. Despite the high overall mask-wearing observed, these differences in mask-wearing within groups are troubling, as the spread of respiratory viruses is much greater when people are in proximity.

Collectively, these findings suggest that relying on direct observations alone may have limitations without additional considerations. First, the results of Study 1 suggest that using logistical considerations, like high-frequency locations, measures may not consistently be sensitive to identifying all meaningful variability in mask-wearing behaviors. This approach may miss additional context or time periods where at-risk behaviors are more likely. Second, using direct observation resources alone may not be sufficient for identifying true high behavioral-risk locations. For example, while mask-wearing percentages were high, increases in COVID-19 cases on campus during that time suggested the observation process did not adequately identify where behavioral risk was highest. Gathering additional information about potential high behavioral-risk locations would better inform direct observation. To address these limitations, collecting self-report survey data on student perceptions of behavioral risk could help identify locations more likely to be observed with at-risk behaviors. One promising strategy is the use of heat mapping, in which participants can visually mark perceived high- and low-mask-wearing areas on a campus map. The result of these analyses could inform where to allocate direct observation resources to verify actual safe and at-risk behavior at these locations.

## Study 2

The purpose of Study 2 was to evaluate whether crowd-sourced perception data could identify high- and low- behavioral-risk locations on a college campus during the COVID-19 pandemic. In this study, students completed a heat mapping survey, in which they indicated locations where they observed high and low levels of mask-wearing. These responses were then aggregated into data heat maps and the resulting visualizations were used to select the three highest and three lowest perceived mask-wearing locations on campus. Direct observation methods were then used to assess actual mask-wearing behavior at these locations. The purpose of the direct observations was to verify the survey results and, therefore, validate the efficacy of survey-based heat mapping for guiding the identification of behavioral risks.

### Hypotheses

Two hypotheses were tested during Study 2. First, we hypothesized that participants would identify distinct special regions as high- and low-mask-wearing locations, with minimal overlap between the two categories (H3). Second, we hypothesized that observed mask-wearing would be significantly lower in high-mask-wearing locations than in low-mask-wearing locations as identified by the survey data and heat map analysis (H4).

### Methods

#### Heat map analysis.

To conduct the heat map analysis, undergraduate students were recruited to complete an online survey administered on the Qualtrics^®^ survey platform. A total of 51 students completed the survey. Students who participated in the survey received.50% extra credit added to their final grade in a course. The following procedures were approved by the university’s Human Research Protection Program (study #20635). The approval period for the study was from February 12^th^, 2020 to February 11^th^, 2023. Participant data collection took place from April 14^th^, 2021 to April 19^th^, 2021. Informed consent was obtained through an electronic response administered through the survey. Specifically, after being provided with an information statement about the study, participants were provided with the following instructions:

I agree to voluntarily participate in this research on consumer decision making (selecting “agree” will begin the research session; if you disagree and do not wish to participate, simply navigate away from this page or close your browser window). If you consent to participate, please click the button with the arrow to continue.

The survey contained a map of the university campus embedded in a Qualtrics^®^ Heat Map Question. The students were first instructed to select up to five places on campus where they see the least mask-wearing during a school day. To indicate a response, the student would click a location on the campus map (e.g., student union, academic building, recreation center). After each response, a visual circle would remain on the location to indicate their selection. Next, students were asked to select up to five places on campus where they see the most mask-wearing during a school day. These data were then generated into data heat maps. Heat maps displayed locations with higher proportions of clicks as red, while locations with fewer clicks were displayed as blue. Two heat maps were created: one displaying reported low mask-wearing locations and one displaying reported high mask-wearing locations.

#### Direct observation procedures.

The top three locations with the lowest and highest reported mask-wearing, as identified in the heat map analyses, were selected for direct observation. Observations were conducted from April 19^th^, 2021 to May 6^th^, 2021. Separate observations were conducted for Study 2 instead of using observation data from Study 1 for two reasons. First, not all of the locations identified in the heat map analysis were observed in Study 1. Secondly, conducting the observations close in proximal time to when the survey was administered could reduce potential history or maturation effects between Studies 1 and 2. Observations were conducted in a similar manner to those in Study 1, with two exceptions. Given the larger surface locations identified for observation in this study (i.e., outside pond and volleyball court), observing when a person walked past a dedicated point was not feasible. For example, on a volleyball court, rapid movements by an individual could result in the person crossing the observation indicator multiple times in quick succession. The subsequent data would then be skewed by that scoring of that one individual multiple times. In contrast, at the outside pond, individuals typically had minimal movement as they would often sit around the pond. Therefore, individuals would not frequently travel between the observation indicator. Therefore, observation sweeps were conducted instead. An observer began looking from left to right and scored mask-wearing during the sweep. To complete one observation, the observer would complete the sweep at several spots in each location. At least 30 minutes were required in between observation sweeps. The other exception is that observations occurred inside the building for locations that were reported as low-mask-wearing locations in this study but were not observed with low-mask-wearing outside in Study 1. Specifically, observation sweeps were completed inside the student union instead of outside, as in Study 1. Observations at the bus stop, academic building, and library used the same observation method described in Study 1.

#### Interobserver agreement.

Interobserver agreements were collected for 24.69% of observations using the same procedure as in Study 1. Mean agreement across all observations was high, averaging 92.83% (range, 53.97%–100%). These data indicate that observation scoring was generally reliable across observers.

### Results

#### Heat map analysis findings.

**Fig 5:**
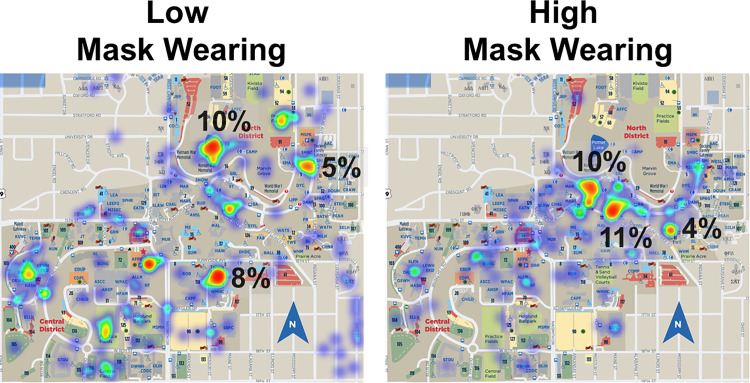
Heat map. Red, yellow, and orange indicate locations with greater proportions of responses. Green, blue, and purple indicate locations with lower proportions of responses. Percentages of all responses are displayed for the top three locations selected.

Fig 5 contains the results of the survey and heat map analysis. Locations reported with low mask-wearing were an outside pond on campus, volleyball courts, and the student union. Locations identified with high mask-wearing were a bus stop in the center of campus, an academic building, and a library. Notably, locations with low mask-wearing were not reported when students were asked to identify high mask-wearing. These results demonstrate differential responses in identifying low and high mask-wearing locations.

**Fig 6:**
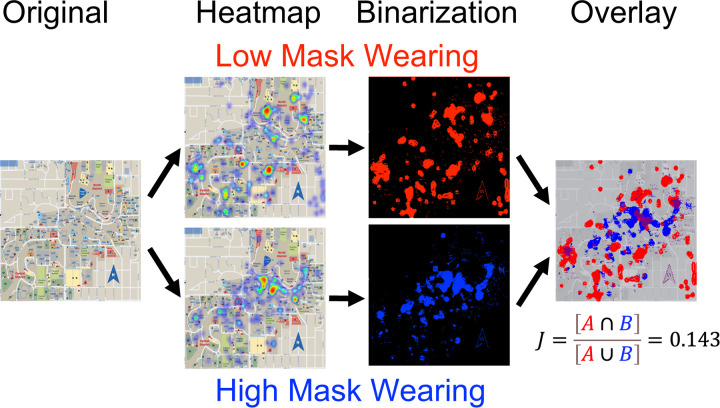
Heatmap binarization and overlay comparison. Visual summary of the spatial analysis pipeline, from the original map (left-most panel), to heatmap generation from survey clicks (middle-left panel), binarization (middle-right panel), and color-coded overlay (right-most panel), which yielded a Jaccard index of 0.143 indicating limited spatial overlap between low (red) and high (blue) mask-wearing regions (see text for details).

To compare spatial distributions of perceived mask-wearing behavior, binary heatmap masks were created from the original full-color heatmap images (corresponding to low-mask and high-mask conditions (see “Original” and “Heatmap” panels [left-panels] in Fig 6). The process began with the generation of RGBA heatmap overlays from kernel density estimates of participant click data. Each image, corresponding to either the low-mask or high-mask condition, was converted from RGBA to HSV color space using the OpenCV library [11] in Python.

Pixels were classified into binary masks based on color characteristics. Pixels with HSV values corresponding to yellow (Hue: 20–35°, Saturation ≥ 100, Value ≥ 100) or black (Value < 50) were classified as background and excluded (i.e., coded as black, 0). All other pixels—typically representing mid-to-high click density in the heatmaps (e.g., red, pink, magenta, or blue regions)—were classified as foreground and retained (i.e., coded as white, 255). This binary classification resulted in one binary image per condition (see the “Binarization” panel in Fig 6).

To enhance visual interpretability, white pixels in the binary low-mask image were recolored as solid red (RGB: 255, 0, 0), and white pixels in the binary high-mask image were recolored as solid blue (RGB: 0, 0, 255). Both recolored binary masks were overlaid onto one another using the PIL.Image.alpha_composite function from the Python Imaging Library. This resulted in a composite image wherein (see right-most panel in Fig 6):

- Red areas indicated regions uniquely associated with perceived low mask-wearing.
- Blue areas indicated regions uniquely associated with perceived high mask-wearing.
- Purple areas (overlapping red and blue) indicated locations identified in both conditions.

The final overlay visualization provided a qualitative spatial comparison of behavioral perceptions across the campus. To quantify the degree of spatial overlap between the perceived mask-wearing conditions, a Jaccard index (also known as the Intersection over Union [12]) was computed using the jaccard_score function from the sklearn.metrics module in Python [13]. Binary masks for the low-mask and high-mask conditions were derived from the respective RGBA overlays (see “Binarization” panels in Fig 6). For each pixel, a value of 1 indicated a non-background classification (i.e., red or blue pixel with α > 0), and a value of 0 indicated background.

Each binary mask was flattened into a one-dimensional vector, allowing for pairwise comparison across all pixels. The Jaccard index (*J*) was then computed as:


$$J=\frac{[A\cap B]}{[A\cup B]}=0.143$$

where *A* is the set of pixels marked in the low-mask binary image, and *B* is the set of pixels marked in the high-mask binary image. The resulting Jaccard index (*J*) was 0.143, indicating that only 14.3% of the non-background pixels were shared between the two conditions. This suggests a substantial spatial distinction in participants’ perceptions of where mask-wearing was observed as low versus high.

To assess whether the spatial overlap between the low-mask and high-mask condition maps was smaller than would be expected by chance, we conducted a permutation analysis of the Jaccard index. The Jaccard index was calculated as the ratio of the intersection to the union of two binary masks created from the respective spatial click data (*J* = 0.143). To evaluate the statistical significance of this observed index, we generated a null distribution of Jaccard indices using 10,000 random permutations. For each iteration, we randomly selected the same number of active pixels (i.e., pixels marked as foreground) as observed in the original low-mask and high-mask binary masks, drawing from the total number of pixels in the image (N = 640,000). This ensured that the permutation preserved the spatial density of each condition while randomizing their locations. The permutation process was implemented in Python 3.11 using NumPy (v1.26) for random sampling and logical operations, and the jaccard_score function from scikit-learn’s sklearn.metrics module (v1.4) for computing the observed Jaccard index. The seed was fixed for reproducibility (np.random.seed(42)). Across all 10,000 permutations, none produced a Jaccard index (*J*) equal to or lower than the observed value (0.143), resulting in a permutation-based *p*-value of 1.000. This indicates that the spatial overlap between perceived low and high mask-wearing regions was significantly lower than expected by chance, given the number of active pixels in each condition.

These findings provide support for H3, which predicted that participants would identify distinct special regions on campus as high- and low-risk for mask-wearing behaviors. The low Jaccard index and subsequent premutation analysis suggest minimal spatial overlap between areas identified as low-mask wearing and high mask-wearing, and that this observed separation was highly unlikely to occur by chance.

#### Direct observation findings.

**Fig 7:**
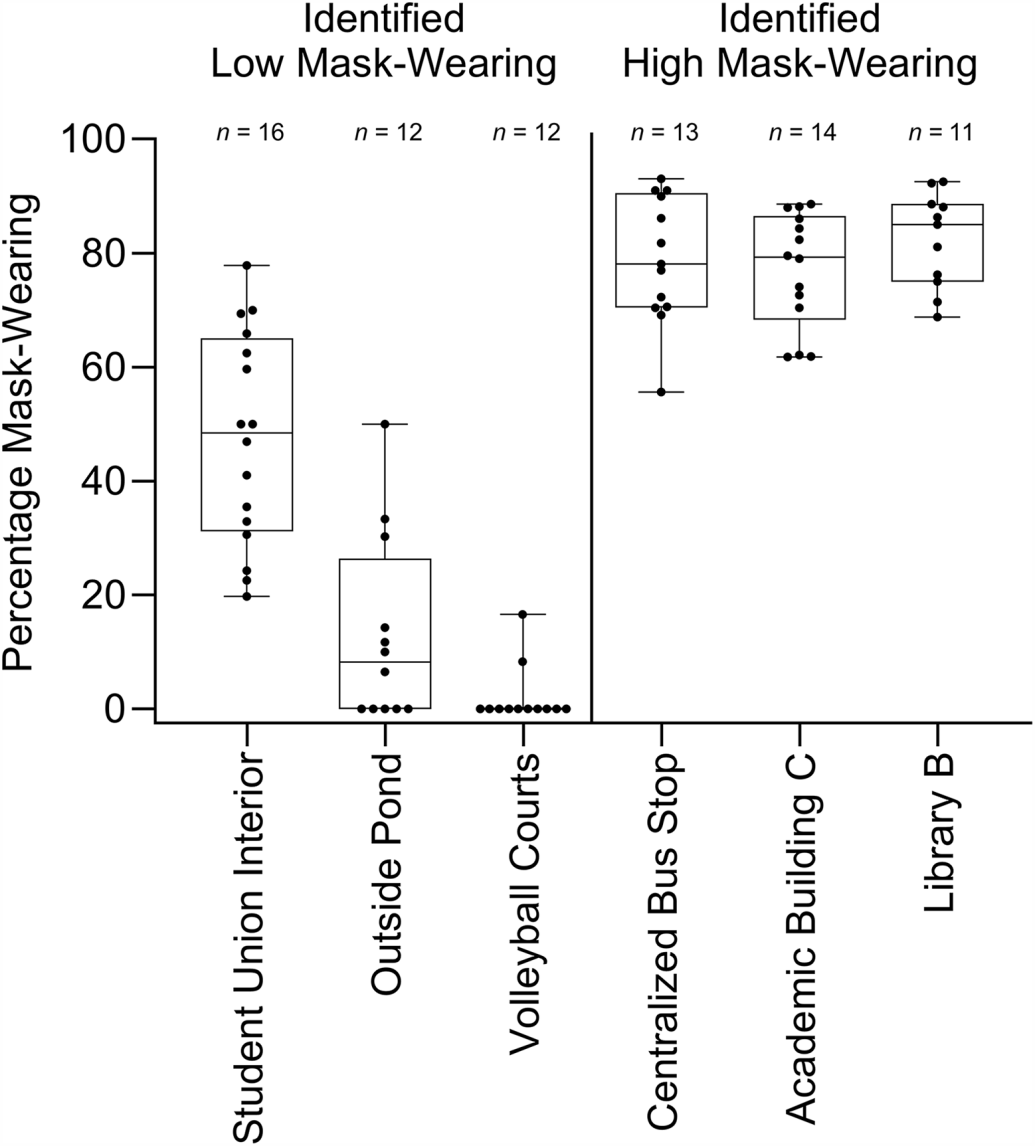
Percentage mask-wearing across heat map locations. Box plot whiskers indicate the minimum and maximum data. The beginning and end of the box plot indicate the 25th and 75th percentile values. The horizontal line within the box plot indicates the median. N represents the number of 30 min observations conducted at that location.

**Fig 8:**
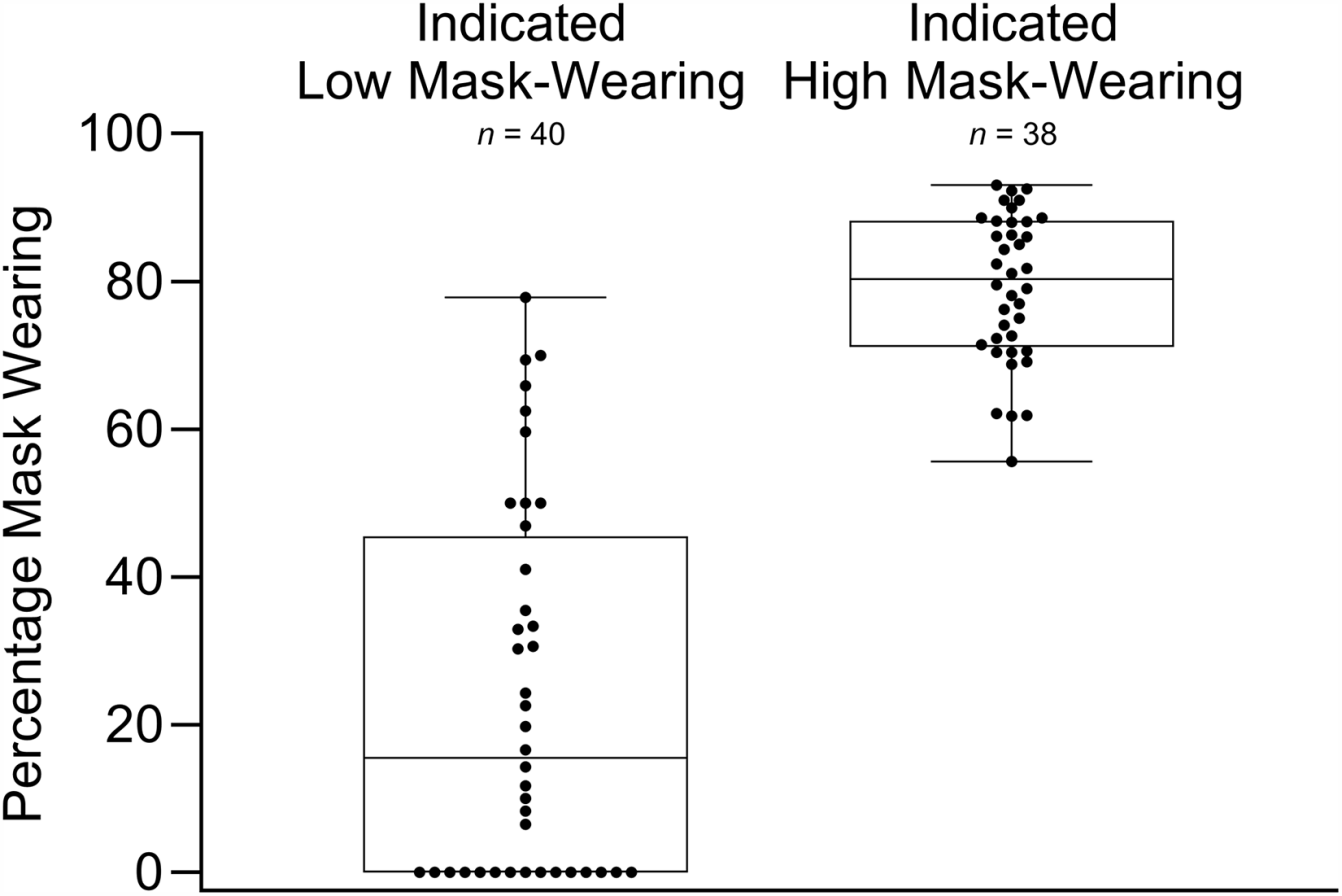
Percentage mask-wearing across indicated low and high mask-wearing locations. Box plot whiskers indicate the minimum and maximum data. The beginning and end of the box plot indicate the 25th and 75th percentile values. The horizontal line within the box plot indicates the median. N represents the number of 30 min observations conducted at those location.

Direct observations were then conducted at the locations identified in the heat map analysis. Over 3 weeks, 78 observations were completed. This resulted in 39 hours of observation and 3,756 behaviors observed. Fig 7 contains each observation across reported low and high mask-wearing locations and the percentage of mask-wearing observed. Results from direct observation revealed that locations identified as low mask-wearing during surveying had lower mask-wearing percentages than locations reported as having high mask-wearing. Specifically, mask-wearing in locations reported as low mask-wearing was low, averaging 23% (range, 0%–78%). In contrast, mask-wearing in locations reported as high mask-wearing was high, averaging 79% (range, 56%–93%). To assess whether mask-wearing differed significantly between locations identified as having high and low mask-wearing, a T-test analysis was conducted using GraphPad Prism® (version 10.1.2). Prior to selecting an appropriate test, the data were assessed for normality. In addition to reviewing distribution plots for normality, and QQplots, a Shapiro-Wilk test assessed whether the data were normally distributed. Although the locations identified as high mask-wearing passed the Shapiro-Wilk test for normality, the locations identified as high-risk did not (*W* = .8497, *p* < .0001) and were therefore not assumed to be normally distributed. Therefore, a non-parametric Mann-Whitney U test was used. Mask-wearing percentages at reported low mask-wearing locations (*Mdn* = 15.45) were significantly lower than those locations reported as high mask-wearing (*Mdn* = 80.29), *W* = 37, *p* < .0001. Fig 8 contains the aggregated data used in the analysis. These results support H4, which predicted that mask-wearing would be significantly lower in locations identified as having low mask-wearing by the survey and heat map analysis compared to those identified as having high mask-wearing.

### Study 2 discussion

The results of Study 2 support the utility of heat mapping as a practical tool for identifying behavioral risk locations on a college campus. Consistent with H3, participants identified spatially distinct low and high mask-wearing locations. The minimal overlap between these two categories demonstrated that participants reliably distinguished between areas of perceived behavioral risk. Additionally, subsequent direct observation data validated the heat map results and supported H4. Specifically, observed mask-wearing was significantly lower in survey locations identified as low mask-wearing compared to locations identified as high mask-wearing. This correspondence between perceived and actual mask-wearing behaviors provides evidence that crowd-sourced heat maps can predict behavioral risk locations.

Overall, these findings demonstrate that heat mapping offers a valid and resource-efficient method for identifying where at-risk behaviors are likely to occur. This approach can more effectively guide the allocation of direct observation efforts and intervention strategies to mitigate the spread of infectious diseases.

## General discussion

The current studies evaluated methods for identifying behavioral risk during a public health crisis on a college campus. A combination of direct observation, crowd-sourced survey data, and heat map visualizations was used to identify at-risk behaviors across a college campus. Across two studies, we used direct observation, surveys, and heat map analyses to (a) identify general patterns of mask-wearing behavior, (b) evaluate whether heat mapping could efficiently identify both low and high mask-wearing locations, and (c) verify those results through direct observation of mask-wearing.

Study 1 demonstrated through direct observation that mask-wearing was generally high and mostly consistent across campus locations during the COVID-19 pandemic. These results are consistent with prior campus observation research in the United States with indoor and outdoor mask-wearing policies [8]. However, mask-wearing was found to be less likely when individuals were within a group rather than alone. This group-status pattern aligns with social norm research suggesting that mask-wearing can be influenced by the behavior of others [4,6]. Despite the observed high mask-wearing observed in Study 1, an increase in COVID-19-related illnesses at the college during that time suggested that high behavioral risk locations were not adequately identified.

Study 2 addressed this limitation by using a survey to gather data about perceptions of locations with low and high mask-wearing. These data were aggregated into data heat maps. Participants identified spatially distinct locations perceived as having low and high mask-wearing. Direct observation validated these findings by demonstrating that locations identified as low mask-wearing had actual low mask-wearing, and locations identified as high mask-wearing had high mask-wearing. These findings extend prior campus heat-mapping work by Gelino, Salzer, Harsin, Naudé, Gilroy, and Reed [10] which asked respondents to identify only where the target behavior was typically seen the most. By asking respondents to identify both low and high mask-wearing locations, we observed differential responses between the two categories. These results suggest that responses were not simply driven by where people congregate (i.e., density). Together, these findings support heat mapping as a practice and resource-efficient way to identify potential low and high behavioral risk locations during health epidemics.

Determining where behavioral risk is likely to occur through heat map analyses can inform policies and procedures to reduce the likelihood of risk. Resources can then be appropriately allocated to these high-risk locations. One practical strength of this approach is its accessibility: heat maps can be easily embedded into surveys administered via platforms like Qualtrics^®^, enabling intuitive click-based input from respondents. This simplicity enhances both participation rates and face validity—participants can visualize their own experiences with risk locations, which in turn produces actionable data. Institutions could implement this method periodically or during times of heightened concern to generate crowdsourced risk indicators across a campus. These visual data outputs can then be used to triage observational resources, conduct follow-up location-specific surveillance, or guide targeted behavioral interventions (e.g., signage, peer prompts, or monitoring). As such, this method offers a scalable, rapid-response tool for identifying and mitigating behavioral risks in dynamic public health contexts. The sampling methods used in these studies required minimal time and observers were trained quickly. The benefit of the additional direct observation is that risk mitigation efforts (e.g., policy and procedure implementation) can be evaluated for effectiveness.

There were several limitations across these studies to note. First, the sample size used for the heat mapping survey was small compared to the full university population. While the survey findings were supported by direct observation, future research should assess the minimum number of responses required to generate stable and representative heat maps. Second, neither of these studies evaluated the effects of an intervention to mitigate potential risk or increase mask-wearing. Future studies could extend this work by using heat mapping and direct observation to assess the impact of interventions implemented to reduce at-risk behavior and increase safe behavior. Finally, the methods demonstrated here could be extended to other health-related behaviors beyond mask-wearing (e.g., physical distancing, hand washing) and to organizational safety contexts (e.g., personal protective equipment).

In summary, these studies suggest that heat mapping and direct behavioral observation can be effective strategies for identifying where safe and at-risk health behaviors occur. Once identified, these locations can be monitored to evaluate the effects of interventions and additional resources implemented to minimize risk. In addition, differences in mask-wearing by group status suggest the importance of considering social norms when assessing behavioral risks. Using heat maps to identify locations and direct observations to verify and quantify behavioral risks provides a practical framework for risk identification on college campuses during pandemics and epidemics.
